# Simulating and Forecasting the COVID-19 Spread in a U.S. Metropolitan Region with a Spatial SEIR Model

**DOI:** 10.3390/ijerph192315771

**Published:** 2022-11-27

**Authors:** Faizeh Hatami, Shi Chen, Rajib Paul, Jean-Claude Thill

**Affiliations:** 1 Department of Geography and Earth Sciences, University of North Carolina at Charlotte, Charlotte, NC 28223, USA; 2 Department of Public Health Sciences, University of North Carolina at Charlotte, Charlotte, NC 28223, USA; 3 School of Data Science, University of North Carolina at Charlotte, Charlotte, NC 28223, USA

**Keywords:** SEIR model, spatial SEIR model, approximate Bayesian computation, mobility, spatial dependence, temporal variability, epidemic model

## Abstract

The global COVID-19 pandemic has taken a heavy toll on health, social, and economic costs since the end of 2019. Predicting the spread of a pandemic is essential to developing effective intervention policies. Since the beginning of this pandemic, many models have been developed to predict its pathways. However, the majority of these models assume homogeneous dynamics over the geographic space, while the pandemic exhibits substantial spatial heterogeneity. In addition, spatial interaction among territorial entities and variations in their magnitude impact the pandemic dynamics. In this study, we used a spatial extension of the SEIR-type epidemiological model to simulate and predict the 4-week number of COVID-19 cases in the Charlotte–Concord–Gastonia Metropolitan Statistical Area (MSA), USA. We incorporated a variety of covariates, including mobility, pharmaceutical, and non-pharmaceutical interventions, demographics, and weather data to improve the model’s predictive performance. We predicted the number of COVID-19 cases for up to four weeks in the 10 counties of the studied MSA simultaneously over the time period 29 March 2020 to 13 March 2021, and compared the results with the reported number of cases using the root-mean-squared error (RMSE) metric. Our results highlight the importance of spatial heterogeneity and spatial interactions among locations in COVID-19 pandemic modeling.

## 1. Introduction

The contribution of spatio-temporal analysis to the understanding of COVID-19 infections and deaths is by now well documented [1,2,3,4]. Not all regions have been impacted with the same rates of infections and deaths. Striking temporal variations were also recorded at both national and regional levels. In early 2020, New York was the epicenter for COVID-19 infections in the US, and then that shifted gradually to Southern states around the July 4th weekend; as Fall approached, the intensity of the disease moved to the Dakotas, and lastly, in January 2022 the Northeast experienced the highest number of new cases of the entire pandemic [5]. The global spread of the disease is linked to international and transregional travel and to different variants of the virus. Local factors such as policies on non-pharmaceutical interventions (e.g., social distancing, mask mandates, school and business closures, etc.), population density and movement patterns, propensity to travel internationally, vaccine availability and uptake rates, and quality of the healthcare infrastructure feature among the core factors of local variations in the spread of the disease and in its prevalence. It is because these local factors condition community transmission, risk of exposure, and community vulnerability to the virus that they are so important at the local level, and are then amplified across the globe through complex patterns of human spatial interactions.

Given the importance of localized scales, the objective of this study was to simulate and predict the spread of COVID-19 in a multi-county metropolitan region with an epidemic model that explicitly accounts for the spatial and temporal heterogeneities of disease spread. Predictions are made over a four-week forecasting horizon and compared with a series of other forecasting models. Four-week ahead predictions are a common practice in the literature on COVID-19 predictive analytics. For instance, the U.S. Centers for Disease Control and Prevention (CDC) report four-week ahead forecasts from a number of leading infectious disease teams around the world [6].

The spatio-temporal analysis of infectious diseases can be broadly classified into three main groups. In one approach, disease clusters and hotspots are assessed using ecological modeling [2,3]; in the second approach, we study the mechanisms in which the hosts and their movement in space and time contribute to disease transmission; the third technique resorts to machine learning and deep learning methods to tease out the role of space and time in predicting the spread of the disease [7,8]. Ecological models are used often on aggregated data to understand the spatial and spatio-temporal variations in disease spread in terms of prevalence and incidence rates with respect to explanatory variables [9]. These models should not be used for causal inference. Mechanistic models have been extensively applied to characterize various epidemiological processes (e.g., transmission, recovery, vaccination, disease-induced death, etc.), parameterize these critical processes, and provide projections of disease dynamics [10,11]. In particular, mechanistic compartmental models are usually expressed as an array of coupled ordinary differential equations (ODEs), Partial differential equations (PDEs), or stochastic differential equations (SDEs) of various processes [12]. The most commonly applied compartmental model is the susceptible–exposed–infectious–recovered (SEIR) model, with the four compartments representing the host population’s transitioning epidemiological states. The susceptible population is the proportion of the entire population that is capable of contracting the pathogen. The exposed compartment is the population that has been exposed to the pathogen but is not yet infectious. Infectious populations are those who have been exposed to the pathogen and have the capability to further transmit the pathogen. Finally, recovered populations are those who are not infectious any more as a result of recovery with immunity or mortality [12]. Over the course of almost a century, SEIR-type compartmental models have been the paradigm of infectious disease modeling. Conducting a systematic review of 72 publications on COVID-19 predictive models, Shankar et al. [13] found that over 70% of models were in the SEIR group. In another review paper, Rahimi et al. [14] reported that SEIR models are in the top three most common COVID-19 outbreak modeling methods. SEIR-type models have been commonly applied in modeling COVID-19 epidemics and in capturing its complexities, incorporating external factors such as non-pharmaceutical interventions (NPIs) and vaccination [15]. The SEIR-type compartment model is relatively easy to understand, formulate, analyze, and extend to accommodate a wide range of disease-related processes. Important metrics such as the basic reproduction number (*R*_0_) and the effective reproduction number (*Re*) can be derived analytically or numerically from the compartment model to evaluate the severity of the pandemic and the effectiveness of interventions, and attempt to forecast the future behavior of the disease.

Modeling frameworks incorporating machine learning or deep learning method leverage the flexibility of these analytical techniques to predict spatio-temporal dynamics. Istaiteh et al. [16] used four machine learning models, including convolutional neural network (CNN), artificial neural network (ANN), long short-term memory (LSTM) and autoregressive integrated moving average (ARIMA) to forecast COVID-19 cases. This study provided forecast for cases over the next 7 days for all countries worldwide. Applying three machine learning methods of hidden Markov chain model (HMM), hierarchical Bayes model, and long short-term-memory model (LSTM), Tian et al. [17] predicted COVID-19 cases in six countries and used the root-mean-square error (RMSE) to evaluate their prediction performance. Nikparvar et al. [18] used LSTM, a type of recurrent neural network, to predict the number of COVID-19 cases and deaths for 4 weeks and for the entire United States at the fine resolution of counties. Zeroual et al. [19] used five deep learning methods to predict COVID-19 new and recovered cases in six countries. They used recurrent neural networks (RNNs) including LSTM, bidirectional LSTM (BiLSTM), gated recurrent units (GRUs), and variational autoencoder (VAE).

Since SEIR-type models are based on ODEs, they usually do not handle spatial processes (e.g., host movement across space) and spatial heterogeneity (e.g., varying disease-related parameters across space such as different transmission coefficients, rates of recovery and death, etc.) well. Nevertheless, today’s human movement patterns are strikingly distinct and more prominent than a century ago when the SEIR model was originally developed. Failure to incorporate modern movement patterns based on spatial heterogeneities may result in unrealistic predictions [20]. Therefore, new methodologies, such as spatial SEIR models, have been developed to address and accommodate the critical spatial component in infectious disease dynamics explicitly. Several new SEIR-based models have recently been advanced that alleviate the limitations of earlier generations of SEIR models to be more in tune with the patent realities of highly mobile and heterogenous contemporary societies. This is the approach we followed in this study.

Spatial SEIR models generally incorporate two or more spatially connected and interacting populations, each having their own parameters related to disease processes (e.g., within-population transmission rate, recovery rate, death rate in the specific population, etc.). Within each population, the disease dynamics can be characterized by the traditional SEIR model as if the population is isolated (e.g., no interactions among populations). More importantly, these populations have interactions (inflow and outflow via commuting) that lead to new transmissions. Intensity or rate of interactions will influence potential transmission, thus changing the overall disease transmission dynamics within and among populations.

In this study, we used a spatial SEIR model to simulate the number of COVID-19 cases and forecast their four-week ahead values in ten counties of the Charlotte–Concord–Gastonia Metropolitan Statistical Area (MSA). In this model, the number of cases was forecasted for all locations in one model, simultaneously. The spatial interactions between different locations were incorporated into the model to enhance the model’s accuracy of simulations and predictions. In addition, due to the impacts of external factors on COVID-19 transition dynamics, a set of covariates were included in this model, including the sociodemographic factors, mobility, county-level political leaning, COVID-19 vaccination coverage, and intervention policies. The model’s predictive performance is evaluated by computing the root-mean-square error (RMSE) between model predictions and reported values.

## 2. Materials and Methods

### 2.1. Study Area

**Figure 1:**
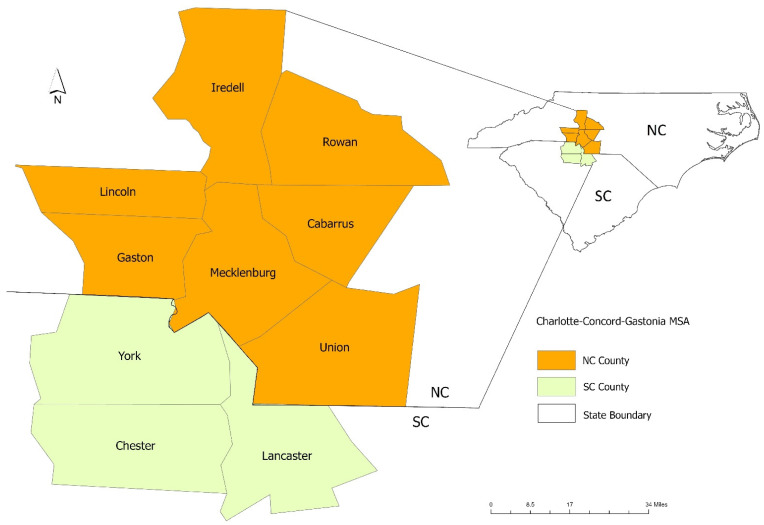
Map of the Charlotte–Concord–Gastonia MSA.

We studied the spatio-temporal dynamics of COVID-19 in the Charlotte–Concord–Gastonia Metropolitan Statistical Area (MSA). This region is one of the largest MSAs in the Southeastern U.S. It comprises a total of 10 counties (7 in North Carolina and 3 in South Carolina) with an estimated total population over 2.6 million in 2021 [21] (Figure 1). Its population has grown by 16% from 2010 to 2021; during this period, population growth reached 22% in Mecklenburg County, North Carolina’s second most populous county [22]. As the most important commuting destination in the MSA, Mecklenburg County has experienced 32% of growth in employment in 10 years (from 697,231 in 2010 to 923,259 in 2020) [23]. It constitutes the employment center of the MSA as about 60% of commuting trips end in this county [24]. Other counties trail Mecklenburg County both in population and employment, and operationally have a residential function within the MSA interlaced with a few outlying business centers.

In addition to fast population and economic growth, the Charlotte area has been experiencing transformative urban development and growth in two major forms relevant to the spread of COVID-19 pandemic: (1) high-intensity suburban developments in the outer parts of the city; and (2) high density and mixed developments in the inner parts of the city. Compact urban developments in the inner parts include the vertical developments in the central areas, infilling in the older neighborhoods around the central city and transit-oriented developments along the transit corridors [25]. Land use transportation plans have been developed to integrate the land developments with a rapid transit system along transit corridors. In addition to the extensive highway system, the LYNX light rail service has enhanced connectivity between different parts of the county and beyond [26].

The county’s large population size, rapid growth with high socio-economic disparity, fluid human movement, and its dominance of the entire MSA make this region vulnerable to large epidemics such as the current COVID-19.

### 2.2. Data Retrieval and Preprocessing

Data for the cumulative counts of COVID-19 cases and deaths in a daily temporal resolution were sourced from the Center for Systems Science and Engineering (CSSE) at Johns Hopkins University [27]. Data were collected from 29 March 2020 to 13 March 2021 for each of the 10 counties in our study area, and then cleaned and preprocessed for fitting the spatial SEIR prediction model (Section 2.3). Data for the following four weeks were used for prediction purposes based on the fitted model. We restricted our analysis to this study period mainly for three reasons: (1) We wanted to study one type of variant of COVID-19, Delta variant (B.1.617.2) started interfering around 20 May 2021, and later became the dominant variant. Thus, we restricted our study prior to arrival of Delta variant; (2) Data monitoring in the regions under study gradually declined as severity of the cases declined and vaccines became more widely available; (3) Finally, beyond 31 March 2021, all nonpharmaceutical restrictions in the region were lifted. During data preprocessing, it was found that some cumulative cases and deaths had smaller values than on the previous day. This issue is resolved by replacing the incorrect value by the value of the previous day, thus ensuring monotonically increasing cumulative cases and deaths. Daily incident cases and new deaths were obtained from the cleaned daily cumulative data through differencing values between two consecutive days. Then, 7-day moving averages of new daily incident cases and new deaths were calculated using a centered moving average method. Finally, daily moving averages of incident cases and new deaths were aggregated to weekly counts. Weekly aggregates were consistent with the Morbidity and Mortality Weekly Report (MMWR) weeks [28].

In addition to the COVID-19 case and death data, covariates critical to COVID-19 dynamics were collected and incorporated in the prediction model: sociodemographic factors such as population density, age and race, mobility, county-level political leaning, COVID-19 vaccination coverage, and intervention policies. These covariates were structured in two groups of time-variant and time-invariant variables, respectively. Time-invariant data were used to capture spatial heterogeneity in this study (i.e., differences between spatial entities, counties), while time-variant variables were used to capture spatio-temporal heterogeneity. In addition, time-variant variables were modeled with a 2-week lag to account for the COVID-19 incubation period [29].

Sociodemographic factors of race, population density, and age distribution were sourced from the SimplyAnalytics database [30], drawing primary data from the US Census Bureau American Community Survey. Mobility patterns are one of the most important covariates of the COVID-19 pandemic [18,31,32]. In order to capture the impact of the interactions between populations in different counties, mobility data were collected from different sources and incorporated in the prediction model. The daily time spent at different places such as home, work, public transit, grocery stores, and parks was obtained from Google COVID-19 Community Mobility Reports [33]. Missing data were imputed by values of the previous day. Daily values were aggregated to weekly values based on the same epidemiological calendar of MMWR. We also used Apple Mobility [34] data, which include the percentage change in mobility on a daily basis, as a reference to cross-validate other mobility data sources. Apple Mobility daily values were aggregated to weekly values for counties following the same approach mentioned earlier. In addition, we collected the total number of individuals’ visits to points of interest (POIs) from the SafeGraph’s Monthly Patterns dataset [35]. SafeGraph collects mobility data from a variety of resources such as mobile phone GPS and provides these data through the SafeGraph COVID-19 Data Consortium [36]. In this dataset, the total tally of daily visits to POIs is available by Census Block Group. In this study, Census Block Group level data were aggregated to counties, and daily visits were aggregated to weekly visits for each county based on the epidemiological calendar.

Another important societal factor that is able to influence perception of the risk of COVID-19 and behavioral responses (e.g., whether or not one is willing to comply with various interventions) is the political leaning of the places [37]. For this purpose, we captured the differences between counties in the voting results of the 2020 presidential elections. Data were collected from the North Carolina State Board of Elections [38] for North Carolina counties, and from the South Carolina Election Commissions [39] for South Carolina counties. Then, the ratio of votes for the Democratic candidate over the Republican candidate was calculated and incorporated into the prediction model.

Given the importance of COVID-19 vaccination, especially its impact on the third and largest wave in COVID-19 cases in 2021, county-level vaccination coverage with at least one dose was obtained for North Carolina counties from the North Carolina Department of Health and Human Services [40]. Historical vaccination data are available for North Carolina counties; however, for South Carolina, only the cumulative data are available. As a result, the weekly counts of vaccinated people with at least one dose for SC counties were estimated on the population-weighted vaccination counts in NC counties. Then, the weekly counts were aggregated to cumulative values to account for the continuous impact of vaccinations over time.

In addition, several other policy-related variables were used; for example, the mandates on face covering starting in June 2020 [41], school shutdowns starting in August 2020. Wintery atmospheric temperature between October 2020 and February 2021 is used as an environmental factor. Descriptive statistics of important variables are summarized in Table 1.

The preprocessed COVID-19 cases were used to construct the data model and the mentioned covariates are used to build the exposure model component of the overall spatial SEIR model in this study (detailed in Section 2.3). Another important component of this spatial model is the distance model (also known as the spatial process model), which specifies exposure probabilities as a linear combination of so-called ‘spatial weight matrices’. These matrices capture the spatial structure of the units of observation; specifically, they measure the geographic proximity (or conversely, their geographic separation of distance) on a pairwise basis. In this study, they were based on the length of the shared boundary between counties and on the inverse of the pairwise distance between the centroids of all 10 counties, using the Cartographic Boundary Files-Shapefile [42]. The preliminary analysis led to the conclusion that such a combination of spatial structures provided a more comprehensive characterization of spatial interaction effects and a more effective handling of disease diffusion across the geographic space than either of these two matrices alone. The two spatial weight matrices were unweighted in the distance model.

### 2.3. Modeling Framework and Model Structure

Brown et al. [43] developed a stochastic spatial SEIR model fitted to data using the approximate Bayesian computational algorithm [44]. It models the dynamics of the epidemic over time at multiple spatial locations. Equation (1) shows the mathematical formulation of the model with four compartments (S, E, I, R), and transitions into new compartments (S*, E*, I* and R*). New transitions indicated by an asterisk occur over discrete times (_i_) and discrete locations (_j_). πij^(RS)^, πij^(SE)^, πj^(EI)^ and πj^(IR)^ show the probabilities of transitions between R and S, S and E, E and I, and I and R, respectively.

S_i+1_ = S_i_ − E*_i_ + S*_i_ ⋯ S*_ij_ ~ binom(R_ij_, π_ij_^(RS)^) 
E_i+1_ = E_i_ − I*_i_ + E*_i_ ⋯ E*_ij_ ~ binom(S_ij_, π_ij_^(SE)^)
I_i+1_ = I_i_ − R*_i_ + I*_i_ ⋯ I*_ij_ ~ binom(E_ij_, π_j_^(EI)^)
R_i+1_ = R_i_ − S*_i_ − R*_i_ ⋯ R*_ij_ ~ binom(I_ij_, π_j_^(IR)^) (1)

where the exposure probabilities {π_ij_^(SE)^} are modeled using exposure-related covariates (such as mobility, vaccinations, population demographics, non-pharmaceutical interventions (NPIs) and weather) and spatial weight structures. The spatial structure of the population is incorporated into the exposure probability as shown in Equation (2). In this equation, D_z_ denotes n-by-n spatial weight matrices, and Ῥ denotes the associated autocorrelation parameters.

(2) $$\;\pi_{\mathrm{ij}}^{\left(\mathrm{SE}\right)}=1-\exp\;(\{-\eta_{i}-\sum_{z=1}^{Z}\;{Ῥ}_{z}(D_{z}\;\eta_{i}){{\}}_{j}}^{\mathrm{hi}})\;$$

π_ij_^(SE)^ in Equation (2) indicates the probability of transitioning a susceptible individual in location s_j_ at time t_i_ into the exposed population.

The probabilities of transitioning from E to I, and from I to R, are explained in Equation (3), in which γ denotes the transition ratio parameter and h_i_ denotes a temporal offset allowing for irregularly spaced time points. More detailed descriptions of the model can be found in Brown et al. [43].

π_i_^(EI)^ = 1 − exp(− h_i_γ_(EI)_) π_i_^(IR)^ = 1 − exp(− h_i_γ_(IR)_) (3)

The ABSEIR *R* package [43] was used to simulate the SEIR model. As a stochastic spatial SEIR model, the overall model is composed of several modules: data model, exposure model, reinfection model, distance model, transition priors, initial value container, and sampling control. After each individual module has been developed, all modules are fed into the spatial SEIR model to simulate the number of reported case series. The data model component describes the input data and their relationship with the SEIR model. A matrix of COVID-19 cases with rows denoting the temporal dimension and columns denoting different locations is fed into the data model. In addition, other parameters such as the matrix type (“identity”, “overdispersion”, or “fractional”), and compartment (I* or R*), were included in the data model. In the data model, it needs to be specified whether the input data (number of cases) are in cumulative format or indicate new cases in each time point. The exposure model component describes the actual epidemic process including various covariates. The reinfection model component indicates whether transitions from recovered (R) to susceptible (S) compartments are allowed in the model or not. The transition priors component specifies the prior transition probabilities from E to I, and from I to R compartments. These two transition priors capture the latency period and infectious period of the dynamics. The distance model describes the spatial network structure of the population as it conditions the interactions among counties in the MSA. The distance model is composed of a list of square, symmetric spatial weight matrices. Each square matrix indicates the pairwise relationships between studied locations. The pairwise relationship can be defined in different ways. For example, it can show the straight-line distance between location centroids, or whether two locations are neighbors or not. The initial value container is constructed based on the total population, number of cases and deaths, and COVID-19 tests at the beginning of the study period, in each county. Lastly, sampling control specifies the approximate Bayesian computation (ABC) algorithm and other sampling properties. Details of the model can be found in the ABSEIR GitHub repository (https://github.com/grantbrown/ABSEIR, accessed on 15 November 2020) by Brown et al. [43].

Once the complete model is constructed and fitted, COVID-19 cases are predicted for up to four weeks ahead for dates between 20 March 2021 to 10 April 2021. Then, model performance (goodness of fit) is evaluated by computing the root-mean-square error (RMSE) between model predictions and reported values. Note that each of the 10 counties in this study will have its own, county-specific model parameters and RMSEs. We developed different versions of the model by including different specifications of covariates (i.e., inclusion of certain combinations of covariates), and optimal models are identified by comparing RMSEs of competing models at each county. The stability of our models’ performance is tested by running the same models on another 4-week period from 17 April 2021 to 8 May 2021. The results of this 4-week period were also compared with the ensemble model as a benchmark.

In order to assess the enhancement made by the inclusion of the spatial interactions’ characteristics in the SEIR model, we also developed comparable models without the spatial interactions. These nonspatial models simultaneously estimate COVID-19 cases for all counties without taking into account their geographic connections. In these models, counties were assumed to be isolated from each other (spatial weight matrices were null).

In addition, we further evaluated the performance of our fitted spatial SEIR models by comparison with models developed in the extant literature. Specifically, we compared our model outputs with an ensemble prediction model implemented by Reich Lab’s COVID-19 Forecast Hub in collaboration with CDC [45]. This ensemble model takes the predicted values of COVID-19 cases and deaths from over 50 international research groups on a weekly basis, and calculates the arithmetic mean and median to construct the ensemble. The COVID-19 Forecast Hub’s ensemble model is a good benchmark to evaluate the performance of our model. The predictive performance of our model on the alternate period from 17 April 2021 to 8 May 2021 was also compared with the ensemble model as a benchmark.

## 3. Results

Multiple spatial SEIR models were constructed based on different specifications for the exposure model component, i.e., with different combinations of covariates/factors. Only the prediction results of three models with best goodness-of-fit measures are reported here. The covariates in each of these models are listed in Table 2.

The three models are reported as each of them performed better than the others in some of the counties. In the other words, no single model performs consistently well across all 10 counties in this study. The spatial variability in model performance indicates that particular spatial and/or spatio-temporal covariates impact COVID-19 dynamics differently in different locations.

**Figure 2:**
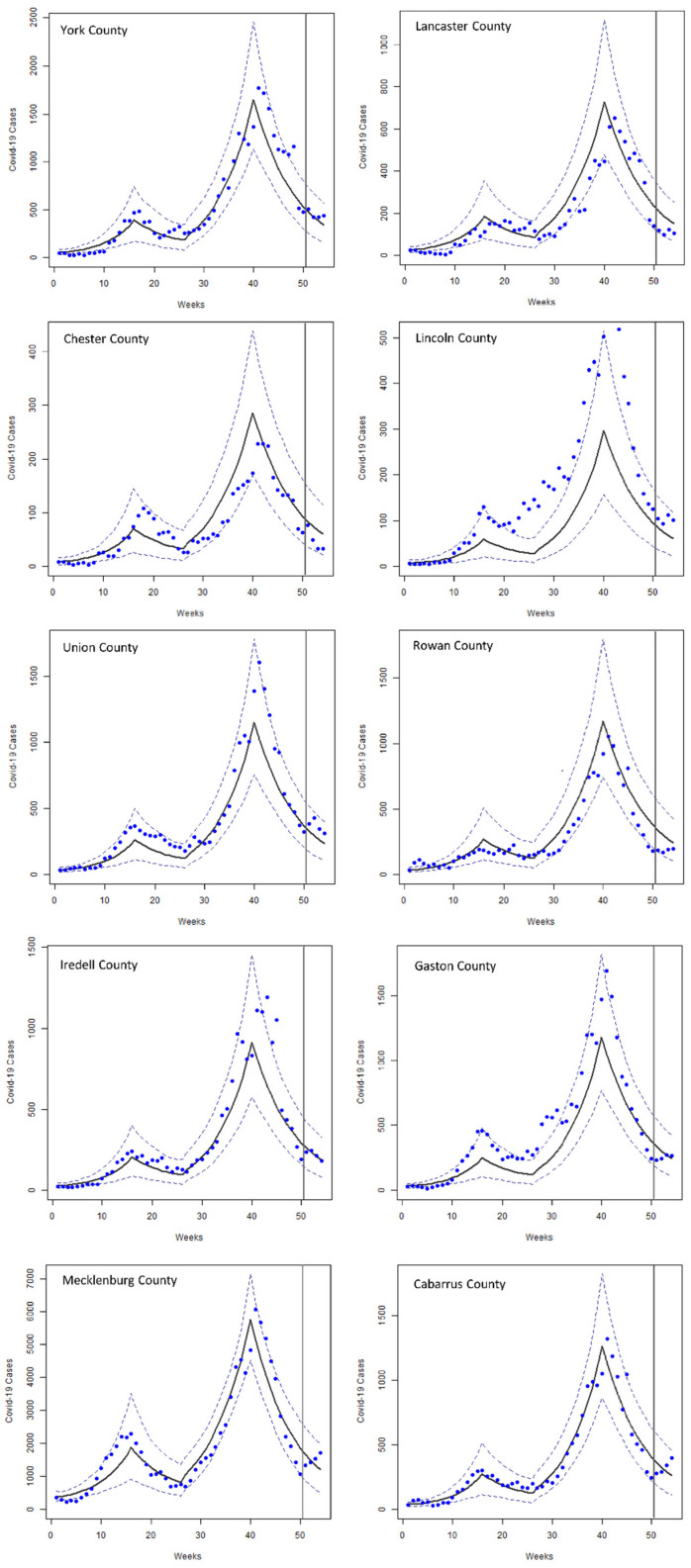
Reported historical (blue dots) COVID-19 cases and cases simulated with model 2 (black line) in 10 counties of the study area, including the last 4 weeks not used for model fitting. The 5% confidence interval is shown with dashed lines. The last 4 weeks to the right of the gray vertical lines show the forecasted number of cases.

Bayes factor, the ratio of the acceptance between two models, is used to compare the overall performance of the three optimal models [46]. As reported in Table 2, all values of the Bayes factor are greater than 3 in model 2, indicating moderate to strong evidence for model 2’s overall better performance than the other two models. On the other hand, none of the factors exceeds 3 in model 3, and only one exceeds 3 in model 1. Taken together, these results suggest that model 2 outperforms the other models. Figure 2 shows fitting and prediction results of model 2 as the overall best model.

Table 3 shows the 4-week ahead prediction of COVID-19 cases with each model between 20 March 2021 to 10 April 2021, as well as the reported cases for benchmarking purposes. With this information, the RMSE values can be calculated for our spatial SEIR model and similarly for the ensemble model from CDC [45]. These are reported in Table 4. Using the ensemble model as a benchmark, we find that our spatial SEIR model performs better than the benchmark model in 8 of the 10 counties (Cabarrus, Gaston, Iredell, Mecklenburg, Union, Rowan, Lancaster and York), weaker in just one county (Lincoln), and on par in Chester County. To compare the overall predictive performance of each model in all counties with the overall performance of the benchmark ensemble model, one overall weighted RMSE value is calculated for each model across all counties and weighted by the county population size (Table 5). We also calculated an overall unweighted RMSE. All of the spatial SEIR models developed in this study perform drastically better in predicting 4-week ahead cases than the benchmark ensemble across all counties.

The three spatial models are also compared with their nonspatial counterparts to establish the specific contribution of the spatial dimension on the modeling of the spread of COVID-19. Integrating the spatial and spatio-temporal dynamics across counties has substantially improved the predictive performances of all specifications of the SEIR model. Specifically, we find that the average 4-week RMSE across all 10 counties has improved by 47%, 31% and 80% in models 1, 2 and 3, respectively, compared with the nonspatial counterparts. There is latent spatial heterogeneity in the spread of COVID-19, which transpires through the predictive performance of the instances of spatial SEIR model as no specification performs better than the others in all 10 counties. Model 1 performs better than other models in three counties; model 2 better than other models in five counties, and model 3 better than other models in two counties. Hence, the overall RMSE values (Table 5) largely hide these differences. However, we note that the weighted RMSE identifies model 1 as the best performer due to the overwhelming population size of Mecklenburg County over other counties, while the unweighted RMSE value puts models 1 and 2 on an equal footing.

Finally, we test the stability of the predictive performance of models over time. For this purpose, we run the same model specifications for another 4 weeks (from 17 April 2021 to 8 May 2021). We have found that our fitted models perform consistently well across prediction periods compared with the benchmark ensemble model.

## 4. Discussion

We have developed a spatial SEIR model to fit the COVID-19 epidemic dynamics and implemented it to predict the number of 4-week ahead cases. SEIR models have been one of the most commonly used methods for studying epidemic dynamics. However, original SEIR models have no explicit spatial component, and cannot adequately capture potential spatial processes and variability across different locations. Spatial SEIR, on the other hand, takes into account the heterogeneity of epidemics across different locations. It also captures the spatial interactions (e.g., mixing via movement and mobility) among these locations.

The spread of COVID-19 is simulated and predicted for future weeks in the 10 counties of the Charlotte–Concord–Gastonia MSA. The relative positions of Mecklenburg County and of the surrounding counties in the MSA, and their respective social determinants of health (e.g., employment rates) and landscape structure in this broader geographic context, are generative of meaningful spatial heterogeneities and lead to strong interactions between these counties including commuting trips, non-work trips, and mobility of goods and services.

Metropolitan areas have been particularly impacted during the COVID-19 pandemic because of spatial interactions and heterogeneity within and between locations in the metropolitan areas. According to the results of this study, the spatial SEIR model proves effective at accounting for these complicated spatial interactions and variations to forecast the future COVID-19 epidemic dynamics. Our model outperforms similarly specified SEIR models by a large margin because it explicitly accounts for spatial context and spatial heterogeneity and for the existence of heterogeneous interactions between constituting spatial parts of the study region. Extensive research in spatial analysis of urban and regional systems [47] has considerably advanced the state-of-the-art in this area. The present study points that this is also the case with population health and with the spread of infectious diseases such as the COVID-19 pandemic. The epidemic dynamics in different counties are highly influenced by the spatio-temporal heterogeneity of external covariates. Model performance varies across counties, which indicates the important role of external covariates and of their spatio-temporal heterogeneity in the spread of COVID-19 in a large, heterogeneous metropolitan region. In our research, when the structure of spatial interactions operationalized by two spatial weight matrices was controlled for during the model fitting process (in effect, assuming that counties are compartmentalized and that there are no interactions between them), it was determined that the goodness-of-fit and predictive power of the SEIR model were significantly downgraded. Hence, the structure of the spatial environment matters as far the spatio-temporal pattern of the incidence of COVID-19 is concerned.

Several studies have addressed the spatial patterns and heterogeneities of COVID-19 in SEIR-type models. For example, Hou et al. [48] treated spatial heterogeneities in inter-county modeling of COVID-19 in Wisconsin using business movement patterns, age, and race as covariates. Embedding a network-cluster-based approach in human mobility flow-augmented stochastic SEIR models, they estimated a region-specific *Re*. They used an Ensemble Kalman Filter approach to fit their proposed models to the data obtained from the Wisconsin Department of Health Service. In another study, Lawson and Kim [49] used a Bayesian space–time SIR model for estimating and predicting county-level COVID-19 infections and deaths in South Carolina. Region-specific model parameters were modeled using intrinsic conditional autoregressive (ICAR) priors. Chiang et al. [50] used the Hawkes process with spatio-temporal covariates based on county-level Google mobility data and demographics for estimating incidences, deaths, and basic reproduction numbers across the 48 contiguous US states via an expectation–maximization algorithm. Kinsey et al.’s [51] Bucky model used a stratified SEIR model based on age groups and geographic locations to estimate case rates, deaths, and healthcare burden. In a more recent study, emphasizing the geographic heterogeneity of pandemic dynamics, Macias et al. [52] proposed a Lagrangian-SEIR-based model to predict the COVID-19 epidemic in the Mexican state of Jalisco. Their approach incorporated a Lagrangian movement model that captures population movements within and among regions. Gopalakrishnan et al. [53] emphasized the impact of spatial granularity on COVID-19 forecasting results using different unit areas of state, county cluster and county; however, they did not take into account the spatial interactions between these areal units in their compartmental forecasting model. Liu and Li [54] proposed a multi-group SEIR model taking into account the spatial heterogeneity through incorporating spatial diffusion and heterogeneity in the model parameters. In addition, emphasizing on the importance of capturing the spatial interactions in SEIR models, Rajuladevi et al. [55], studied the impact of seeding or initializations in a network of different countries. They found that the effect varies over different countries. Our work expanded this literature by exemplifying the application of spatial SEIR on a metropolitan region and assessed the spatial variations in models’ predictive power. Our work opens avenues for future research on fitting various models with different covariates in different regions under spatial SEIR set up with appropriate penalization [56] and variable selection [57,58] techniques. This study has several limitations. While spatial SEIR represents the future of epidemic modeling and forecasting, questions remain to be investigated to realize the full potential of this sub-family of models. First, it remains to be determined what epidemics benefit to be modeled with an explicit representation of spatial heterogeneity and interactions (also known as spatial dependence). To what extent would different models of space (especially of the spatial weight matrix) for different types of viral infections enhance the performance of the models? Spatial analysis and spatial econometrics have made great strides over the past decade in some respects with a number of modeling frameworks, but not with SEIR-type models to this day.

Second, given the known behaviors of the COVID-19 epidemic, we see the need to develop models at a higher spatial resolution than the current county-level to explicitly represent community-level transmission, and even multi-level models with nested spatial representations that match the range of diffusion processes between place-based populations. Third, we envision that machine learning can greatly enhance the performance of spatial SEIR models by optimizing model parameters that fit the local geographic contexts. In addition, many diseases have concurrent variants/strains such as COVID-19 (e.g., transition period between Alpha and Delta, and Delta and Omicron variants in the U.S.), which substantially influence epidemic dynamics. With broader and more accurate genomic and serosurveillance, we will expand spatial SEIR models to explicitly incorporate multiple variants/strains and their composition in different regions.

## 5. Conclusions

In this study, we developed a spatial SEIR model capable of simulating and predicting the number of 4-week ahead COVID-19 cases in the Charlotte–Concord–Gastonia MSA. The analysis concluded the spatial SEIR is effective at predicting the spatio-temporal dynamics of the pandemic by explicitly accounting for spatial heterogeneity and spatial interaction between counties.

In addition to pathogen biology, COVID-19 pandemic is influenced by various external covariates such as host mobility, demographic factors, non-pharmaceutical interventions such as school closures, and pharmaceutical interventions such as vaccinations. As a result, the epidemic dynamics vary across different locations based on variations in these factors. In this study, we developed different models incorporating a variety of external covariates and relevant datasets, such as face mask intervention policies, school closings, seasonal atmospheric temperature drops, population density, vaccinated population with at least one dose, presidential election voting ratio, percentage change in mobility from Apple Mobility dataset, and change in time spent at work from the Google mobility reports dataset. We found that spatio-temporal heterogeneity of these external covariates is a strong predictor of the epidemic dynamics. We have identified both the time variant and time invariant covariates that are critical to local (county-specific) COVID-19 epidemic dynamics. Among all competing models that we have developed, three models perform better than others, based on RMSE values as the model evaluation metric. The variation in model performances across counties harks back to the influential impact of external covariates and their spatio-temporal heterogeneity in COVID-19 diffusion in a diverse metropolitan region.

Our analysis has shown that the spatial SEIR model is effective at accounting for the complicated spatial interactions and variations within and between locations in the metropolitan areas to forecast the future COVID-19 epidemic dynamics. Our models outperformed equivalent SEIR models that rule out spatial dependence and spatial interaction between counties. We also compared and evaluated our model with the CDC’s ensemble model. Our spatial SEIR models performed on a par with, or even better than, the benchmark ensemble model, showing the importance of incorporating high-resolution, location-specific covariates to further enhance model predictability at the metropolitan scale.

With regard to the tremendous socioeconomic costs of the COVID-19 outbreak, it is a high priority to be able to predict the epidemic dynamics beforehand. This information can be used to inform timely and effective policy such as pharmaceutical and non-pharmaceutical interventions. Our spatial SEIR model developed for Charlotte–Concord–Gastonia MSA can be readily extended to other regions (especially metropolitan areas with high spatio-temporal heterogeneity) to effectively characterize and predict the COVID-19 epidemic dynamics.

## Figures and Tables

**Table 1:** Descriptive statistics of variables used in the final exposure model.

Variable	Mean	Std. Dev.	Min	Pctl. 25	Pctl. 75	Max
Google mobility (change in time spent at workplaces (%))	−28.835	8.118	−53.714	−33.893	−22.714	−10.429
Population vaccinated with at least one dose	1028.341	2375.554	0	0	306.25	13162
Apple mobility (average requests for changing directions)	130.352	24.699	53.999	119.248	148.376	180.517
Voting ratio (democrats/republicans)	0.756	0.468	0.361	0.508	0.8	2.094
Population density (per mi2)	515.209	551.934	55.335	273.888	579.502	2097.705
Wintery temperature	0.63	0.48	0	0	1	1
School shutdowns	0.39	0.49	0	0	1	1
Face mask intervention policy	0.28	0.45	0	0	1	1

**Table 2:** Three retained spatial SEIR models: specification and approximate Bayes factors.

	Model Specification	Approximate Bayes Factor		
		Model 1	Model 2	Model 3
Model 1	Face mask intervention policy School shutdowns Wintery temperature Population density Vaccinated population with at least one dose	1.0	0.2	3.1
Model 2	Face mask intervention policy School shutdowns Wintery temperature Presidential election voting ratio Vaccinated population with at least one dose Percentage change in mobility (Apple Mobility dataset)	5.6	1.0	17.3
Model 3	Face mask intervention policy School shutdowns Wintery temperature Presidential election voting ratio Vaccinated population with at least one dose Change in time spent at work (Google mobility reports dataset)	0.3	0.1	1.0

**Table 3:** Four-week predicted cases (PC) and reported cases (RC) across 10 counties in the MSA.

		County									
		Cabarrus	Gaston	Iredell	Lincoln	Mecklenburg	Rowan	Union	Chester	Lancaster	York
PC Model 1	W1	284	251	226	64	1677	265	299	72	207	454
	W2	256	227	204	58	1515	240	270	65	188	410
	W3	232	205	184	53	1372	217	244	59	170	370
	W4	210	186	167	47	1241	196	221	53	153	336
PC Model 2	W1	364	337	261	85	1665	338	328	84	209	472
	W2	326	301	234	76	1492	303	294	75	188	423
	W3	292	270	210	68	1340	271	263	67	168	379
	W4	262	243	188	61	1203	243	236	60	151	340
PC Model 3	W1	181	170	132	36	1661	160	174	41	113	269
	W2	162	151	118	32	1484	143	156	37	101	240
	W3	144	135	105	29	1328	127	139	33	90	214
	W4	130	121	94	26	1189	114	125	29	81	192
RC	W1	277	233	236	104	1328	184	383	77	120	503
	W2	289	245	244	93	1436	166	424	50	100	419
	W3	343	270	213	112	1532	190	346	33	123	420
	W4	396	268	183	101	1724	196	313	32	106	440

**Table 4:** Four-week ahead prediction RMSE values of the three variants of the spatial SEIR model and of the ensemble model for each county.

County	Cabarrus	Gaston	Iredell	Lincoln	Mecklenburg	Rowan	Union	Chester	Lancaster	York
Spatial SEIR—Model 1	110	54	26	48	311	56	111	18	70	63
Spatial SEIR—Model 2	86	60	14	32	326	113	90	26	70	56
Spatial SEIR—Model 3	184	115	108	72	332	54	220	19	21	218
Ensemble	174	92	102	23	882	86	148	18	22	139

**Table 5:** RMSE values of prediction models over 4 weeks and all counties.

	Model 1	Model 2	Model 3	Ensemble
RMSE- average weighted by county population	172	177	224	440
RMSE- unweighted average	87	87	134	169

## Data Availability

Data used in this study will be provided upon request.
